# Caregivers’ burden analytics: combining variables from patients with dementia and their caregivers

**DOI:** 10.1186/s12877-025-06284-y

**Published:** 2025-08-14

**Authors:** Kai-Ming Jhang, Cheng-Chiang Chen, Sih-Yi Wang, Wen-Fu Wang, Shao-Wei Yen, Hsin-Hung Wu

**Affiliations:** 1 Neurological Institute, Changhua Christian Hospital, Changhua, Taiwan; 2 Department of Neurology, Hsinchu Cathay General Hospital, Hsinchu, Taiwan; 3 Department of Business Administration, National Changhua University of Education, Changhua, Taiwan; 4 Department of Information Management, National Changhua University of Education, Changhua, Taiwan; 5 Faculty of Education, State University of Malang, Malang, East Java, Indonesia

**Keywords:** Patients with dementia, Behavior and psychiatric symptoms of dementia, Neuropsychiatric inventory, Caregiving burden, Dementia collaborative care team, Apriori algorithm

## Abstract

**Background:**

Caregivers caring for patients with dementia could experience depressive symptoms, distress from patients’ behavioral symptoms, and physical morbidities. Factors associated with a caregiving burden are complex and vary with time because burden is a subjective measure influenced by physical, economic, and psychosocial strain and has an interaction among caregiver resources, vulnerabilities, and care demands. The purpose of this study was to elucidate the association of patients’ and caregivers’ factors especially the severity of behavior and psychiatric symptoms of dementia (BPSD) and specific activities of daily living items impairment with a moderate or even severe caregiving burden.

**Methods:**

A cross-sectional study was conducted with 585 patients with dementia who were cared for informal caregivers and were managed by the dementia collaborative care team at Changhua Christian Hospital in Taiwan. Variables from patients with dementia included age, gender, type of dementia, clinical dementia rating, feeding, toilet use, bathing, mobility, getting lost, and Neuropsychiatric Inventory, whereas variables from caregivers consisted of age, relation to the patient, marital status, employment, type of primary care, frequency of care, and caregiving burden. The Apriori algorithm was used to find the association of patients’ and caregivers’ factors and a moderate or even severe caregiving burden when the minimum support and confidence were set to 1% and 85%, respectively, along with lift > 1.

**Results:**

One hundred and fifty rules were found for caregivers with a moderate to severe burden, and these rules can be further summarized into twenty general rules. To further explore the rules containing severe burden, the minimum confidence was reduced from 85 to 65%. Sixteen rules were found with a severe caregiving burden, and these rules were concluded into one general rule. When the caregiver was a spouse who solely cared for the male patient with moderate Alzheimer’s disease (AD), the caregiver had a severe burden. Moderately severe BPSD was associated with a high caregiving burden than specific activities of daily living domain dysfunctions.

**Conclusions:**

The severity of BPSD, severity of cognition, patients’ and caregivers’ gender, relation to the care recipient, employment, and caregiving load were associated with a moderate or severe burden for caregivers who cared for patients with dementia. A severe caregiving burden was occurred when a female caregiver solely took care of her spouse who had moderate AD. Suitable caregiving supports should be provided to female caregivers in order to reduce their caregiving burden.

**Supplementary Information:**

The online version contains supplementary material available at 10.1186/s12877-025-06284-y.

## Background

Dementia has become one of the greatest global challenges for health and social care [1]. Dementia is also a challenging age-related illness for both caregivers and healthcare professionals [2, 3]. Cognitive and functional decline as well as behavior and psychiatric symptoms of dementia (BPSD) generate a considerable burden on caregivers [4–6]. Increased caregiving burden influences the clinical outcomes of both people living with dementia (PLWD) and their caregivers. Higher caregiving burden is associated with worse patient quality of life [7, 8]. Caregivers with a high burden experienced more depressive symptoms and distress from behavioral symptoms of PLWD [9] and also physical morbidities such as poorer self-reported health, preventive health behaviors, cardiovascular functioning, and greater health care utilization [10, 11].

Factors associated with a caregiving burden are complex and vary with time since burden is a subjective measure influenced by physical, economic, and psychosocial strain and has an interaction among caregiver resources, vulnerabilities, and care demands [12]. Previous systematic reviews reported that no uniform model for predicting the trajectory of caregiving burden [13]but severer BPSD and functional decline were factors reported frequently and should be considered as important factors to be associated with a high caregiving burden [2]. Although fewer reports support, types of dementia, caregiver’s socio-demographical and psychological factors, cohabitation, and kinship within the dyads were also possible important factors [2, 13]. A previous cohort study followed 18 months found that patients with Lewy body disease, the severer dementia stage or BPSD, and caregiver depression were associated with a high caregiving burden [4]. 

To delineated caring scenario associated with a high burden for caregivers who care for PLWD, we used the Apriori algorithm, a data miming method, from a multidimensional viewpoint. Our previous study which included patients’ and caregivers’ basic characteristics and care load concluded that when a wife solely took care of her husband living with dementia, who was 75–79 years old and still could walk independently, she would experience a high caregiving burden [6]. Another study further included caregivers’ psychiatric conditions and found the following scenarios were associated with a moderate to severe burden: caregivers expressed any one of the mood (emotional liability, depressive, or anxious) and patients still could walk independently [14]. The presence of specific neuropsychiatric symptoms were associated with varying degrees of caregiving burden in female patients with Alzheimer’s disease, for example, crying spells and aggression led to a moderate to severe burden [15]. In patients with vascular cognitive impairment, an employed female caregiver who took care of her spouse for ≥ 6 days per week and helped with all key activities was likely to experience a moderate to severe burden [16]. 

Severer BPSD and worsen activities of daily living (ADL) function were important factors associated with a high caregiving burden [2]. Their significance may also be different. Machnicki et al. [5] reported that behavioral symptoms had more than two times higher strength of an association than functional decline on caregiving burden. Studies discussing spatial disorientation and caregiving burden were relatively limited. A previous report showed that caregivers experienced significant degrees of psychological disturbances after the incident of getting lost [17]. Our previous reports did not include the severity of BPSD, spatial disorientation, and items of ADL to form caring scenarios. Therefore, the aim of the present study was to elucidate the caring scenario by combining patients’ and caregivers’ factors, especially BPSD severity and specific ADL items impairment, with a moderate or high caregiving burden by using the Apriori algorithm.

## Methods

Patients who were diagnosed with dementia at the memory clinic of Changhua Christian Hospital (CCH) from January 2015 to July 2021 were enrolled in this study. For each patient, the diagnosis of dementia was made by attending physicians at CCH memory clinic and the severity of dementia was evaluated by a clinical psychologist based on the clinical dementia rating (CDR) scale [18]. The attending physicians at CCH memory clinic invited the patients with dementia who still lived in the community and their caregivers to join the case manager-centered dementia collaborative care model [19]. More than 90% of patients diagnosed with dementia were enrolled in the model. The care team consisted of various professionals, including physicians, psychologists, social workers, dieticians, occupational therapists, pharmacists, and nursing case managers. After joining the care model, face-to-face evaluations regarding to ADL function, BPSD, living status and care modes, caring problems, and caregiving burden were conducted every 6 months to identify unmet care needs and provide relevant interventions [20–22]. The care team stopped following up if the patients did not visit the dementia clinic for more than 6 months, refused the assessment, became nursing home residents, or died. For caregiving burden, only the family caregiver who accompanied the patient to the clinic was evaluated. If more than one family caregivers visited, the primary caregiver’s burden was assessed. All data were recorded in the dementia case management system and saved as electronic charts [19]. Initially, there were 2120 patients but only 585 patients (27.6%) had complete data to meet the above requirements from both patients and caregivers for analysis. To better reflect the conditions for patients with dementia and their caregivers in a timely basis, the most recent diagnosis and interview data were used.

In this study, the variables from patients with dementia such as age, gender, type of dementia, CDR, feeding, toilet use, bathing, mobility, getting lost, and Neuropsychiatric Inventory (NPI) were provided in Table 1. Dementia severity was categorized as very mild (CDR = 0.5), mild (CDR = 1), moderate (CDR = 2), and severe (CDR = 3) stages. Dementia subtypes were diagnosed and classified in accordance with different guidelines. AD was corresponded to the National Institute on Aging-Alzheimer’s Association (NIA-AA) [23, 24] whereas the diagnostic criteria for vascular cognitive impairment were from the International Society for Vascular Behavioral and Cognitive Disorders (VASCOG) [25]. The diagnosis of Parkinson’s disease dementia or dementia with Lewy bodies (DLB) followed the Movement Disorder Society-Task force criteria and the fourth consensus report of the DLB Consortium [26, 27]. The functional assessment including bathing, feeding, toilet use, and mobility were created by the CCH dementia center. Some patients with dementia developed ADL behaviors such as defecate indiscriminately, need remind, or even pressurized to perform bathing. Case managers performed the ADL evaluation and provided relevant care plans during the assessment. It was worth to note that there were 12 items in the Neuropsychiatric Inventory questionnaire, and the NPI total score could range from 12 to 144 points, where the NPI total score > 36 was recommended as a cutoff value which was considered in need of antipsychotic drug treatment [28]. That is, this study defined a patient to have moderately severe neuropsychiatric symptoms if the NPI total score was larger than 36. More than 80% of the patients were aged 75 years or more, and nearly two-thirds of the patients were female. More than 60% of the patients were diagnosed by AD, and the CDR range for the majority of the patients (89.6%) were from very mild dementia to moderate dementia. Moreover, more than 90% of the patients had mild NPI score.

**Table 1:** Variables from patients with dementia

Variables		Frequency	Percentage	Data Type
Age	< 65 years old 65–74 years old 75–84 years old ≥ 85 years old	19 92 246 228	3.2 15.7 42.1 39.0	1 2 3 4
Gender	Female Male	379 206	64.8 35.2	0 1
Type of dementia	Alzheimer’s disease Vascular dementia Mixed dementia Dementia with Lewy bodies Parkinson’s disease Alcohol related dementia Frontotemporal degeneration Others	357 98 19 15 35 1 10 50	61.0 16.8 3.2 2.6 6.0 0.2 1.7 8.5	1 2 3 4 5 6 7 8
Clinical dementia rating	Very mild dementia Mild dementia Moderate dementia Severe dementia	173 207 144 61	29.6 35.4 24.6 10.4	0 1 2 3
Feeding	Independent Feed by others On nasogastric tube	538 39 8	91.9 6.7 1.4	0 1 2
Toilet use	Independent Remind needed Defecate indiscriminately Incontinence	483 59 2 41	82.6 10.1 0.3 7.0	0 1 2 3
Bathing	Independent Reminder needed Pressurization needed Partially aid needed Complete dependent	369 23 8 131 54	63.1 3.9 1.4 22.4 9.2	0 1 2 3 4
Mobility	Independent Assistance needed Wheel-bound Bedridden	364 154 65 2	62.2 26.4 11.1 0.3	0 1 2 3
Getting lost	Never getting lost Tend to get lost History of getting lost	465 82 38	79.5 14.0 6.5	0 1 2
Neuropsychiatric inventory (NPI)	NPI score: mild NPI score: moderately severe	539 46	92.1 7.9	0 1

The variables from caregivers were composed of age, relation to the patient, marital status, employment, type of primary care, frequency of care, and caregiving burden as shown in Table 2. It is worth to note that there were five types of primary care in Table 2 consisting of shared caregiving by a caregiver and a (foreign) caretaker and caregiving by a foreign caretaker. The caretaker in shared caregiving could be an either domestic or foreign caregiver. In Taiwan, families were the principal source of support and care for PLWD, enabling them to continue living at home [29]. Due to the shortage of local care labors, recruiting trained or untrained migrant care workers (foreign caretakers) from less developed countries to fill the long-term care workforce in elderly care institutions and private households was the major solution in Taiwan [29, 30]. The trained foreign caretakers were required to undergo at least 90 h of predeparture training including basic skills for housework and elderly care and limited Mandarin-learning before working on a live-in basis to care for PLWD [30]. 

**Table 2:** Variables from caregivers

Variables	Frequency	Percentage	Data Type
Age			
<50 years old	132	22.6	1
50-59 years old	215	36.7	2
60-69 years old	125	21.4	3
≥70 years old	113	19.3	4
Relation to the patient			
Spouse	140	23.9	1
Partner	1	0.2	2
Child	348	59.5	3
Brother/sister	6	1.0	4
Other relative	90	15.4	5
Marital status			
Married	494	84.4	1
Divorce	8	1.4	2
Widow/widower	10	1.7	3
Separate	0	0	4
Cohabitation	1	0.2	5
Single	69	11.8	6
Unknown	3	0.5	7
Employment			
Unemployed or retired	259	44.3	0
Employed	326	55.7	1
Type of primary care			
Sole caregiver	189	32.3	1
Shared caregiving by a caregiver and a (foreign) caretaker	211	36.1	2
Shared caregiving by different relatives	12	2.0	3
Caregiving by a foreign caretaker	87	14.9	4
Other	86	14.7	5
Frequency of care			
1-2 days per week	58	9.9	1
3-5 days per week	59	10.1	2
≥6 days per week	468	80.0	3
Caregiving burden			
Little or no burden	186	31.8	1: applied to a particular burden; and 0: otherwise
Mild to moderate burden	275	47.0	
Moderate to severe burden	106	18.1	
Severe burden	18	3.1	

The caregiving burden was evaluated by the Zarit burden interview (ZBI) with four categories, i.e., little or no burden (0–20 points), mild to moderate burden (21–40 points), moderate to severe burden (41–60 points), and severe burden (61–88 points) [31]. A previous study conducted by Wang et al. [32] has shown that the Chinese version of ZBI was reliable for health status measures. For caregiving burden, Only the family caregiver who accompanied the patient to the clinic was evaluated. If more than one family caregivers who took care of the patient, the primary caregiver’s burden was assessed. When a caregiving burden falls in a particular category, a value of 1 is assigned, and a value of zero is given to the other three categories. The age of the caregivers was distributed uniformly across four age groups, and the majority of caregivers were married (84.4%). Frequency of care ≥ 6 days per week (80.0%), and children (59.5%) and spouses (23.9%) were the major forces to take care of PLWD. Additionally, mild to moderate burden (47.0%) and little or no burden (31.8%) were two major categories for the caregiving burden.

The purpose of this study was to elucidate the association of patients’ and caregivers’ factors especially BPSD severity and specific ADL items impairment with a moderate or even severe caregiving burden. Association ruling mining, which was very popular due to its simplicity to find recurring relationships between/among attributes, was suitable to analyze large databases when the data were from a multidimensional contingency Tables [33–35] The basic concept was to discover interesting associations and correlations between attributes in transactional and relational databases [35]. The Apriori algorithm developed by Agrawal and Srikant [36] used the downward closure property to reduce the size of the explored search space by pruning candidate attributes and could be employed to large databases, much larger that were amenable to other types of analyses [34, 37]. 

Studies such as Jhang et al. [20] and Chen et al. [38] showed that the Apriori algorithm was an effective approach to find care need bundles as well as long-term care services usage bundles for PLWD by revealing statistical association through setting up support, confidence, and lift. The definitions of support, confidence, and lift are summarized below [39–41]. The support (A ⇒ B) evaluates the percentage of transactions containing both A and B in the database as shown in Eq. (1):

1 $$\mathrm{Support}\;\left(\mathrm A\;\Rightarrow\;\mathrm B\right)\;=\;P\left(\mathrm A\cap\mathrm B\right)\;=\;\frac{\mathrm{number}\;\mathrm{of}\;\mathrm{transactions}\;\mathrm{containing}\;\mathrm{both}\;\mathrm A\;\mathrm{and}\;\mathrm B}{\mathrm{total}\;\mathrm{number}\;\mathrm{of}\;\mathrm{transactions}}$$

The confidence (A ⇒ B), depicted in Eq. (2), is to calculate the percentage of transactions containing A and also containing B simultaneously in the database:

2 $$\mathrm{Confidence}\;\left(\mathrm A\;\Rightarrow\;\mathrm B\right)\;=\;P\left(B\vert A\right)\;=\;\frac{\mathrm P\left(\mathrm A\cap\mathrm B\right)}{\mathrm P\left(\mathrm A\right)}\;=\;\frac{\mathrm{number}\;\mathrm{of}\;\mathrm{transaction}\;\mathrm{containing}\;\mathrm{both}\;\mathrm A\;\mathrm{and}\;\mathrm B}{\mathrm{number}\;\mathrm{of}\;\mathrm{transaction}\;\mathrm{containing}\;\mathrm A}$$

Lift evaluates if the correlation of A and B is either independent or dependent as shown in Eq. (3). When a lift value has a value of one, then A and B are independent and no rule is generated containing either event. On the other hand, when a lift value is larger than one, then A and B are dependent positively.

3 $$\mathrm{Lift}\;\left(\mathrm A,\mathrm B\right)\;=\;\frac{\mathrm P\left(\mathrm A\cap\mathrm B\right)}{\mathrm P\left(\mathrm A\right)\mathrm P\left(\mathrm B\right)}$$

The Apriori algorithm in IBM SPSS Modeler 14.1 was employed. Data types for variables from patients with dementia and their caregivers defined by the numerical values were summarized in Tables 1 and 2.. The input variables for antecedents included the variables from both the patients with dementia and their caregivers except for caregiving burden. In contrast, two major types of caregiving burden, i.e., moderate to severe burden and severe burden, were the input variables for consequents. Due to the heterogeneous data, the minimum support and confidence were set to 1% and 85%, respectively, along with lift > 1. With the current settings, no rules containing severe burden were found. To further explore the rules containing severe burden, the minimum confidence was reduced to 65% along with the minimum support of 1% and lift > 1.

## Results

One hundred and fifty rules were found for caregivers with a moderate to severe burden depicted in Table 3, and these rules can be further summarized into twenty general rules. The rules generated were based upon the conditional probabilities as shown in Eq. (2), where notations A and B in the equation represented the antecedents (the variables from both the patients with dementia and their caregivers except for caregiving burden) and the consequents (moderate to severe burden and severe burden), respectively. In addition, each general rule was summarized based upon the similarities of rules in terms of the antecedents and consequents. To better understand how each rule could be interpreted properly, the first rule in the first general rule in Table 3 was illustrated. When the following four elements including “CDR: Moderate dementia”, “Bathing: Independent”, “NPI: Moderately severe”, and “Employment: Employed” were simultaneously met, a caregiver would have a moderate to severe burden. In other words, if the consequents were to be present, the elements in the antecedents should be found at the same time. The first general rule containing 8 rules characterized the caregiver as having a moderate to severe burden when the caregiver was employed who took care of the moderate dementia patient with moderately severe NPI who could bath independently. The second general rule was based on 9 rules which showed that the caregiver had a moderate to severe burden when the caregiver was employed with 50–59 years old who took care of the dementia patient with moderately severe NPI.

**Table 3:** Twenty general rules for caregivers with a moderate to severe burden

Rule no.	Antecedent	No. of Cases	Support (%)	Confidence (%)	Lift
1	CDR: Moderate dementia Bathing: Independent NPI: Moderately severe Employment: Employed	6	1.026	100.0	5.519
	CDR: Moderate dementia Bathing: Independent Mobility: Independent NPI: Moderately severe Employment: Employed	6	1.026	100.0	5.519
	CDR: Moderate dementia Toilet use: Independent Bathing: Independent NPI: Moderately severe Employment: Employed	6	1.026	100.0	5.519
	CDR: Moderate dementia Feeding: Independent Bathing: Independent NPI: Moderately severe Employment: Employed	6	1.026	100.0	5.519
	CDR: Moderate dementia Toilet use: Independent Bathing: Independent Mobility: Independent NPI: Moderately severe Employment: Employed	6	1.026	100.0	5.519
	CDR: Moderate dementia Feeding: Independent Bathing: Independent Mobility: Independent NPI: Moderately severe Employment: Employed	6	1.026	100.0	5.519
	CDR: Moderate dementia Feeding: Independent Toilet use: Independent Bathing: Independent NPI: Moderately severe Employment: Employed	6	1.026	100.0	5.519
	CDR: Moderate dementia Feeding: Independent Toilet use: Independent Bathing: Independent Mobility: Independent NPI: Moderately severe Employment: Employed	6	1.026	100.0	5.519
2	NPI: Moderately severe Age of the caregiver: 50–59 Employment: Employed	8	1.368	87.5	4.829
	Toilet use: Independent NPI: Moderately severe Age of the caregiver: 50–59 Employment: Employed	8	1.368	87.5	4.829
	NPI: Moderately severe Age of the caregiver: 50–59 Caregiver’s marital status: Married Employment: Employed	8	1.368	87.5	4.829
	Feeding: Independent NPI: Moderately severe Age of the caregiver: 50–59 Employment: Employed	8	1.368	87.5	4.829
	Toilet use: Independent NPI: Moderately severe Age of the caregiver: 50–59 Caregiver’s marital status: Married Employment: Employed	8	1.368	87.5	4.829
	Feeding: Independent Toilet use: Independent NPI: Moderately severe Age of the caregiver: 50–59 Employment: Employed	8	1.368	87.5	4.829
	Feeding: Independent NPI: Moderately severe Age of the caregiver: 50–59 Caregiver’s marital status: Married Employment: Employed	8	1.368	87.5	4.829
	Feeding: Independent Toilet use: Independent NPI: Moderately severe Age of the caregiver: 50–59 Caregiver’s marital status: Married Employment: Employed	8	1.368	87.5	4.829
	Gender of the patient: Female Feeding: Independent Toilet use: Independent Getting lost: Never getting lost NPI: Moderately severe Age of the caregiver: 50–59 Caregiver’s marital status: Married Employment: Employed	7	1.197	85.714	4.730
3	Gender of the patient: Female NPI: Moderately severe Type of primary care: Shared caregiving by a caregiver and a (foreign) caretaker	7	1.197	85.714	4.730
	Gender of the patient: Female Getting lost: Never getting lost NPI: Moderately severe Type of primary care: Shared caregiving by a caregiver and a (foreign) caretaker	7	1.197	85.714	4.730
4	Gender of the patient: Female CDR: Moderate dementia Mobility: Independent NPI: Moderately severe	7	1.197	85.714	4.730
	Gender of the patient: Female CDR: Moderate dementia Mobility: Independent Getting lost: Never getting lost NPI: Moderately severe	7	1.197	85.714	4.730
	Gender of the patient: Female CDR: Moderate dementia Toilet use: Independent Mobility: Independent NPI: Moderately severe	7	1.197	85.714	4.730
	Gender of the patient: Female CDR: Moderate dementia Feeding: Independent Mobility: Independent NPI: Moderately severe	7	1.197	85.714	4.730
	Gender of the patient: Female CDR: Moderate dementia Toilet use: Independent Mobility: Independent Getting lost: Never getting lost NPI: Moderately severe	7	1.197	85.714	4.730
	Gender of the patient: Female CDR: Moderate dementia Feeding: Independent Mobility: Independent Getting lost: Never getting lost NPI: Moderately severe	7	1.197	85.714	4.730
	Gender of the patient: Female CDR: Moderate dementia Feeding: Independent Toilet use: Independent Mobility: Independent NPI: Moderately severe	7	1.197	85.714	4.730
	Gender of the patient: Female CDR: Moderate dementia Feeding: Independent Toilet use: Independent Mobility: Independent Getting lost: Never getting lost NPI: Moderately severe	7	1.197	85.714	4.730
5	CDR: Moderate dementia Mobility: Independent NPI: Moderately severe Employment: Employed	7	1.197	85.714	4.730
	CDR: Moderate dementia Toilet use: Independent Mobility: Independent NPI: Moderately severe Employment: Employed	7	1.197	85.714	4.730
	CDR: Moderate dementia Feeding: Independent Mobility: Independent NPI: Moderately severe Employment: Employed	7	1.197	85.714	4.730
	CDR: Moderate dementia Feeding: Independent Toilet use: Independent Mobility: Independent NPI: Moderately severe Employment: Employed	7	1.197	85.714	4.730
6	Gender of the patient: Female NPI: Moderately severe Type of primary care: Shared caregiving by a caregiver and a (foreign) caretaker Frequency of care: ≥ 6 days per week	7	1.197	85.714	4.730
	Gender of the patient: Female Getting lost: Never getting lost NPI: Moderately severe Type of primary care: Shared caregiving by a caregiver and a (foreign) caretaker Frequency of care: ≥ 6 days per week	7	1.197	85.714	4.730
7	Age of the patient: 75–84 Gender of the patient: Female NPI: Moderately severe Age of the caregiver: 50–59	7	1.197	85.714	4.730
	Age of the patient: 75–84 Getting lost: Never getting lost NPI: Moderately severe Age of the caregiver: 50–59	7	1.197	85.714	4.730
	Age of the patient: 75–84 Gender of the patient: Female Getting lost: Never getting lost NPI: Moderately severe Age of the caregiver: 50–59	7	1.197	85.714	4.730
	Age of the patient: 75–84 Gender of the patient: Female Toilet use: Independent NPI: Moderately severe Age of the caregiver: 50–59	7	1.197	85.714	4.730
	Age of the patient: 75–84 Gender of the patient: Female Feeding: Independent NPI: Moderately severe Age of the caregiver: 50–59	7	1.197	85.714	4.730
	Age of the patient: 75–84 Toilet use: Independent Getting lost: Never getting lost NPI: Moderately severe Age of the caregiver: 50–59	7	1.197	85.714	4.730
	Age of the patient: 75–84 Feeding: Independent Getting lost: Never getting lost NPI: Moderately severe Age of the caregiver: 50–59	7	1.197	85.714	4.730
	Age of the patient: 75–84 Gender of the patient: Female Toilet use: Independent Getting lost: Never getting lost NPI: Moderately severe Age of the caregiver: 50–59	7	1.197	85.714	4.730
	Age of the patient: 75–84 Gender of the patient: Female Feeding: Independent Getting lost: Never getting lost NPI: Moderately severe Age of the caregiver: 50–59	7	1.197	85.714	4.730
	Age of the patient: 75–84 Gender of the patient: Female Feeding: Independent Toilet use: Independent NPI: Moderately severe Age of the caregiver: 50–59	7	1.197	85.714	4.730
	Age of the patient: 75–84 Feeding: Independent Toilet use: Independent Getting lost: Never getting lost NPI: Moderately severe Age of the caregiver: 50–59	7	1.197	85.714	4.730
	Age of the patient: 75–84 Gender of the patient: Female Feeding: Independent Toilet use: Independent Getting lost: Never getting lost NPI: Moderately severe Age of the caregiver: 50–59	7	1.197	85.714	4.730
8	NPI: Moderately severe Age of the caregiver: 50–59 Employment: Employed Frequency of care: ≥ 6 days per week	7	1.197	85.714	4.730
	Toilet use: Independent NPI: Moderately severe Age of the caregiver: 50–59 Employment: Employed Frequency of care: ≥ 6 days per week	7	1.197	85.714	4.730
	NPI: Moderately severe Age of the caregiver: 50–59 Caregiver’s marital status: Married Employment: Employed Frequency of care: ≥ 6 days per week	7	1.197	85.714	4.730
	Feeding: Independent NPI: Moderately severe Age of the caregiver: 50–59 Employment: Employed Frequency of care: ≥ 6 days per week	7	1.197	85.714	4.730
	Toilet use: Independent NPI: Moderately severe Age of the caregiver: 50–59 Caregiver’s marital status: Married Employment: Employed Frequency of care: ≥ 6 days per week	7	1.197	85.714	4.730
	Toilet use: Independent Feeding: Independent NPI: Moderately severe Age of the caregiver: 50–59 Employment: Employed Frequency of care: ≥ 6 days per week	7	1.197	85.714	4.730
	Feeding: Independent NPI: Moderately severe Age of the caregiver: 50–59 Caregiver’s marital status: Married Employment: Employed Frequency of care: ≥ 6 days per week	7	1.197	85.714	4.730
	Toilet use: Independent Feeding: Independent NPI: Moderately severe Age of the caregiver: 50–59 Caregiver’s marital status: Married Employment: Employed Frequency of care: ≥ 6 days per week	7	1.197	85.714	4.730
9	Gender of the patient: Female NPI: Moderately severe Age of the caregiver: 50–59 Employment: Employed	7	1.197	85.714	4.730
	Getting lost: Never getting lost NPI: Moderately severe Age of the caregiver: 50–59 Employment: Employed	7	1.197	85.714	4.730
	Gender of the patient: Female Getting lost: Never getting lost NPI: Moderately severe Age of the caregiver: 50–59 Employment: Employed	7	1.197	85.714	4.730
	Gender of the patient: Female Toilet use: Independent NPI: Moderately severe Age of the caregiver: 50–59 Employment: Employed	7	1.197	85.714	4.730
	Gender of the patient: Female NPI: Moderately severe Age of the caregiver: 50–59 Caregiver’s marital status: Married Employment: Employed	7	1.197	85.714	4.730
	Gender of the patient: Female Feeding: Independent NPI: Moderately severe Age of the caregiver: 50–59 Employment: Employed	7	1.197	85.714	4.730
	Toilet use: Independent Getting lost: Never getting lost NPI: Moderately severe Age of the caregiver: 50–59 Employment: Employed	7	1.197	85.714	4.730
	Getting lost: Never getting lost NPI: Moderately severe Age of the caregiver: 50–59 Caregiver’s marital status: Married Employment: Employed	7	1.197	85.714	4.730
	Feeding: Independent Getting lost: Never getting lost NPI: Moderately severe Age of the caregiver: 50–59 Employment: Employed	7	1.197	85.714	4.730
	Gender of the patient: Female Toilet use: Independent Getting lost: Never getting lost NPI: Moderately severe Age of the caregiver: 50–59 Employment: Employed	7	1.197	85.714	4.730
	Gender of the patient: Female Getting lost: Never getting lost NPI: Moderately severe Age of the caregiver: 50–59 Caregiver’s marital status: Married Employment: Employed	7	1.197	85.714	4.730
	Gender of the patient: Female Feeding: Independent Getting lost: Never getting lost NPI: Moderately severe Age of the caregiver: 50–59 Employment: Employed	7	1.197	85.714	4.730
	Gender of the patient: Female Toilet use: Independent NPI: Moderately severe Age of the caregiver: 50–59 Caregiver’s marital status: Married Employment: Employed	7	1.197	85.714	4.730
	Gender of the patient: Female Feeding: Independent Toilet use: Independent NPI: Moderately severe Age of the caregiver: 50–59 Employment: Employed	7	1.197	85.714	4.730
	Gender of the patient: Female Feeding: Independent NPI: Moderately severe Age of the caregiver: 50–59 Caregiver’s marital status: Married Employment: Employed	7	1.197	85.714	4.730
	Toilet use: Independent Getting lost: Never getting lost NPI: Moderately severe Age of the caregiver: 50–59 Caregiver’s marital status: Married Employment: Employed	7	1.197	85.714	4.730
	Feeding: Independent Toilet use: Independent Getting lost: Never getting lost NPI: Moderately severe Age of the caregiver: 50–59 Employment: Employed	7	1.197	85.714	4.730
	Feeding: Independent Getting lost: Never getting lost NPI: Moderately severe Age of the caregiver: 50–59 Caregiver’s marital status: Married Employment: Employed	7	1.197	85.714	4.730
	Gender of the patient: Female Toilet use: Independent Getting lost: Never getting lost NPI: Moderately severe Age of the caregiver: 50–59 Caregiver’s marital status: Married Employment: Employed	7	1.197	85.714	4.730
	Gender of the patient: Female Feeding: Independent Toilet use: Independent Getting lost: Never getting lost NPI: Moderately severe Age of the caregiver: 50–59 Employment: Employed	7	1.197	85.714	4.730
	Gender of the patient: Female Feeding: Independent Getting lost: Never getting lost NPI: Moderately severe Age of the caregiver: 50–59 Caregiver’s marital status: Married Employment: Employed	7	1.197	85.714	4.730
	Gender of the patient: Female Feeding: Independent Toilet use: Independent NPI: Moderately severe Age of the caregiver: 50–59 Caregiver’s marital status: Married Employment: Employed	7	1.197	85.714	4.730
	Feeding: Independent Toilet use: Independent Getting lost: Never getting lost NPI: Moderately severe Age of the caregiver: 50–59 Caregiver’s marital status: Married Employment: Employed	7	1.197	85.714	4.730
10	Age of the patient: ≥ 85 Type of dementia: Alzheimer’s disease Bathing: Independent Relation to the patient: Other relative Employment: Employed Type of primary care: Shared caregiving by a caregiver and a (foreign) caretaker	7	1.197	85.714	4.730
	Age of the patient: ≥ 85 Gender of the patient: Female Type of dementia: Alzheimer’s disease Relation to the patient: Other relative Employment: Employed Type of primary care: Shared caregiving by a caregiver and a (foreign) caretaker	7	1.197	85.714	4.730
	Age of the patient: ≥ 85 Gender of the patient: Female Type of dementia: Alzheimer’s disease Bathing: Independent Relation to the patient: Other relative Employment: Employed Type of primary care: Shared caregiving by a caregiver and a (foreign) caretaker	7	1.197	85.714	4.730
	Age of the patient: ≥ 85 Type of dementia: Alzheimer’s disease Toilet use: Independent Bathing: Independent Relation to the patient: Other relative Employment: Employed Type of primary care: Shared caregiving by a caregiver and a (foreign) caretaker	7	1.197	85.714	4.730
	Age of the patient: ≥ 85 Type of dementia: Alzheimer’s disease Feeding: Independent Bathing: Independent Relation to the patient: Other relative Employment: Employed Type of primary care: Shared caregiving by a caregiver and a (foreign) caretaker	7	1.197	85.714	4.730
	Age of the patient: ≥ 85 Gender of the patient: Female Type of dementia: Alzheimer’s disease Toilet use: Independent Relation to the patient: Other relative Employment: Employed Type of primary care: Shared caregiving by a caregiver and a (foreign) caretaker	7	1.197	85.714	4.730
	Age of the patient: ≥ 85 Gender of the patient: Female Type of dementia: Alzheimer’s disease Feeding: Independent Relation to the patient: Other relative Employment: Employed Type of primary care: Shared caregiving by a caregiver and a (foreign) caretaker	7	1.197	85.714	4.730
	Age of the patient: ≥ 85 Gender of the patient: Female Type of dementia: Alzheimer’s disease Toilet use: Independent Bathing: Independent Relation to the patient: Other relative Employment: Employed Type of primary care: Shared caregiving by a caregiver and a (foreign) caretaker	7	1.197	85.714	4.730
	Age of the patient: ≥ 85 Gender of the patient: Female Type of dementia: Alzheimer’s disease Feeding: Independent Bathing: Independent Relation to the patient: Other relative Employment: Employed Type of primary care: Shared caregiving by a caregiver and a (foreign) caretaker	7	1.197	85.714	4.730
	Age of the patient: ≥ 85 Type of dementia: Alzheimer’s disease Feeding: Independent Toilet use: Independent Bathing: Independent Relation to the patient: Other relative Employment: Employed Type of primary care: Shared caregiving by a caregiver and a (foreign) caretaker	7	1.197	85.714	4.730
	Age of the patient: ≥ 85 Gender of the patient: Female Type of dementia: Alzheimer’s disease Feeding: Independent Toilet use: Independent Relation to the patient: Other relative Employment: Employed Type of primary care: Shared caregiving by a caregiver and a (foreign) caretaker	7	1.197	85.714	4.730
	Age of the patient: ≥ 85 Gender of the patient: Female Type of dementia: Alzheimer’s disease Feeding: Independent Toilet use: Independent Bathing: Independent Relation to the patient: Other relative Employment: Employed Type of primary care: Shared caregiving by a caregiver and a (foreign) caretaker	7	1.197	85.714	4.730
11	Age of the patient: ≥ 85 Gender of the patient: Female CDR: Mild dementia Mobility: Independent Age of the caregiver: 50–59 Caregiver’s marital status: Married Frequency of care: ≥ 6 days per week	7	1.197	85.714	4.730
	Age of the patient: ≥ 85 Gender of the patient: Female CDR: Mild dementia Toilet use: Independent Mobility: Independent Age of the caregiver: 50–59 Caregiver’s marital status: Married Frequency of care: ≥ 6 days per week	7	1.197	85.714	4.730
	Age of the patient: ≥ 85 Gender of the patient: Female CDR: Mild dementia Feeding: Independent Mobility: Independent Age of the caregiver: 50–59 Caregiver’s marital status: Married Frequency of care: ≥ 6 days per week	7	1.197	85.714	4.730
	Age of the patient: ≥ 85 Gender of the patient: Female CDR: Mild dementia Feeding: Independent Toilet use: Independent Mobility: Independent Age of the caregiver: 50–59 Caregiver’s marital status: Married Frequency of care: ≥ 6 days per week	7	1.197	85.714	4.730
12	Age of the patient: ≥ 85 Gender of the patient: Female Bathing: Independent Relation to the patient: Other relative Employment: Employed Type of primary care: Shared caregiving by a caregiver and a (foreign) caretaker	7	1.197	85.714	4.730
	Age of the patient: ≥ 85 Gender of the patient: Female Toilet use: Independent Bathing: Independent Relation to the patient: Other relative Employment: Employed Type of primary care: Shared caregiving by a caregiver and a (foreign) caretaker	7	1.197	85.714	4.730
	Age of the patient: ≥ 85 Gender of the patient: Female Feeding: Independent Bathing: Independent Relation to the patient: Other relative Employment: Employed Type of primary care: Shared caregiving by a caregiver and a (foreign) caretaker	7	1.197	85.714	4.730
	Age of the patient: ≥ 85 Gender of the patient: Female Feeding: Independent Toilet use: Independent Relation to the patient: Other relative Employment: Employed Type of primary care: Shared caregiving by a caregiver and a (foreign) caretaker	7	1.197	85.714	4.730
13	Age of the patient: 75–84 Gender of the patient: Female NPI: Moderately severe Age of the caregiver: 50–59 Relation to the patient: Child	7	1.197	85.714	4.730
	Age of the patient: 75–84 Gender of the patient: Female Getting lost: Never getting lost NPI: Moderately severe Age of the caregiver: 50–59 Relation to the patient: Child	7	1.197	85.714	4.730
	Age of the patient: 75–84 Gender of the patient: Female Toilet use: Independent NPI: Moderately severe Age of the caregiver: 50–59 Relation to the patient: Child	7	1.197	85.714	4.730
	Age of the patient: 75–84 Gender of the patient: Female Feeding: Independent NPI: Moderately severe Age of the caregiver: 50–59 Relation to the patient: Child	7	1.197	85.714	4.730
	Age of the patient: 75–84 Gender of the patient: Female Toilet use: Independent Getting lost: Never getting lost NPI: Moderately severe Age of the caregiver: 50–59 Relation to the patient: Child	7	1.197	85.714	4.730
	Age of the patient: 75–84 Gender of the patient: Female Feeding: Independent Getting lost: Never getting lost NPI: Moderately severe Age of the caregiver: 50–59 Relation to the patient: Child	7	1.197	85.714	4.730
	Age of the patient: 75–84 Gender of the patient: Female Feeding: Independent Toilet use: Independent NPI: Moderately severe Age of the caregiver: 50–59 Relation to the patient: Child	7	1.197	85.714	4.730
	Age of the patient: 75–84 Gender of the patient: Female Feeding: Independent Toilet use: Independent Getting lost: Never getting lost NPI: Moderately severe Age of the caregiver: 50–59 Relation to the patient: Child	7	1.197	85.714	4.730
	Age of the patient: 75–84 Getting lost: Never getting lost NPI: Moderately severe Age of the caregiver: 50–59 Relation to the patient: Child	7	1.197	85.714	4.730
	Age of the patient: 75–84 Toilet use: Independent Getting lost: Never getting lost NPI: Moderately severe Age of the caregiver: 50–59 Relation to the patient: Child	7	1.197	85.714	4.730
	Age of the patients 75–84 Feeding: Independent Getting lost: Never getting lost NPI: Moderately severe Age of the caregiver: 50–59 Relation to the patient: Child	7	1.197	85.714	4.730
	Age of the patient: 75–84 Feeding: Independent Toilet use: Independent Getting lost: Never getting lost NPI: Moderately severe Age of the caregiver: 50–59 Relation to the patient: Child	7	1.197	85.714	4.730
14	Age of the patient: ≥ 85 Bathing: Independent Getting lost: Never getting lost NPI: Moderately severe	7	1.197	85.714	4.730
	Age of the patient: ≥ 85 Bathing: Independent Getting lost: Never getting lost NPI: Moderately severe Frequency of care: ≥ 6 days per week	7	1.197	85.714	4.730
	Age of the patient: ≥ 85 Toilet use: Independent Bathing: Independent Getting lost: Never getting lost NPI: Moderately severe	7	1.197	85.714	4.730
	Age of the patient: ≥ 85 Feeding: Independent Bathing: Independent Getting lost: Never getting lost NPI: Moderately severe	7	1.197	85.714	4.730
	Age of the patient: ≥ 85 Feeding: Independent Toilet use: Independent Bathing: Independent Getting lost: Never getting lost NPI: Moderately severe	7	1.197	85.714	4.730
15	Age of the patient: 75–84 Gender of the patient: Female Feeding: Independent NPI: Moderately severe Relation to the patient: Child	7	1.197	85.714	4.730
	Age of the patient: 75–84 Gender of the patient: Female Feeding: Independent Getting lost: Never getting lost NPI: Moderately severe Relation to the patient: Child	7	1.197	85.714	4.730
	Age of the patient: 75–84 Gender of the patient: Female Feeding: Independent Toilet use: Independent NPI: Moderately severe Relation to the patient: Child	7	1.197	85.714	4.730
	Age of the patient: 75–84 Gender of the patient: Female Feeding: Independent Toilet use: Independent Getting lost: Never getting lost NPI: Moderately severe Relation to the patient: Child	7	1.197	85.714	4.730
16	Age of the patient: ≥ 85 Toilet use: Independent Bathing: Independent Getting lost: Never getting lost NPI: Moderately severe Frequency of care: ≥ 6 days per week	7	1.197	85.714	4.730
	Age of the patient: ≥ 85 Feeding: Independent Bathing: Independent Getting lost: Never getting lost NPI: Moderately severe Frequency of care: ≥ 6 days per week	7	1.197	85.714	4.730
	Age of the patient: ≥ 85 Feeding: Independent Toilet use: Independent Bathing: Independent Getting lost: Never getting lost NPI: Moderately severe Frequency of care: ≥ 6 days per week	7	1.197	85.714	4.730
17	Bathing: Independent Mobility: Independent Getting lost: Never getting lost NPI: Moderately severe Age of the caregiver: 50–59	7	1.197	85.714	4.730
	Gender of the patient: Female Mobility: Independent Getting lost: Never getting lost NPI: Moderately severe Age of the caregiver: 50–59	7	1.197	85.714	4.730
	Gender of the patient: Female Toilet use: Independent Mobility: Independent NPI: Moderately severe Age of the caregiver: 50–59	7	1.197	85.714	4.730
	Gender of the patient: Female Feeding: Independent Mobility: Independent NPI: Moderately severe Age of the caregiver: 50–59	7	1.197	85.714	4.730
	Toilet use: Independent Bathing: Independent Mobility: Independent Getting lost: Never getting lost NPI: Moderately severe Age of the caregiver: 50–59	7	1.197	85.714	4.730
	Feeding: Independent Bathing: Independent Mobility: Independent Getting lost: Never getting lost NPI: Moderately severe Age of the caregiver: 50–59	7	1.197	85.714	4.730
	Gender of the patient: Female Toilet use: Independent Mobility: Independent Getting lost: Never getting lost NPI: Moderately severe Age of the caregiver: 50–59	7	1.197	85.714	4.730
	Gender of the patient: Female Feeding: Independent Mobility: Independent Getting lost: Never getting lost NPI: Moderately severe Age of the caregiver: 50–59	7	1.197	85.714	4.730
	Gender of the patient: Female Feeding: Independent Toilet use: Independent Mobility: Independent NPI: Moderately severe Age of the caregiver: 50–59	7	1.197	85.714	4.730
	Feeding: Independent Toilet use: Independent Bathing: Independent Mobility: Independent Getting lost: Never getting lost NPI: Moderately severe Age of the caregiver: 50–59	7	1.197	85.714	4.730
	Gender of the patient: Female Feeding: Independent Toilet use: Independent Mobility: Independent Getting lost: Never getting lost NPI: Moderately severe Age of the caregiver: 50–59	7	1.197	85.714	4.730
18	Age of the patient: ≥ 85 Toilet use: Independent Age of the caregiver: 50–59 Caregiver’s marital status: Married Type of primary care: Sole caregiver	7	1.197	85.714	4.730
	Age of the patient: ≥ 85 Toilet use: Independent Age of the caregiver: 50–59 Caregiver’s marital status: Married Type of primary care: Sole caregiver Frequency of care: ≥ 6 days per week	7	1.197	85.714	4.730
	Age of the patient: ≥ 85 Feeding: Independent Toilet use: Independent Age of the caregiver: 50–59 Caregiver’s marital status: Married Type of primary care: Sole caregiver	7	1.197	85.714	4.730
	Age of the patient: ≥ 85 Feeding: Independent Toilet use: Independent Age of the caregiver: 50–59 Caregiver’s marital status: Married Type of primary care: Sole caregiver Frequency of care: ≥ 6 days per week	7	1.197	85.714	4.730
19	Type of dementia: Alzheimer’s disease Bathing: Independent Mobility: Independent Getting lost: Never getting lost NPI: Moderately severe Age of the caregiver: 50–59	7	1.197	85.714	4.730
	Gender of the patient: Female Type of dementia: Alzheimer’s disease Mobility: Independent Getting lost: Never getting lost NPI: Moderately severe Age of the caregiver: 50–59	7	1.197	85.714	4.730
	Gender of the patient: Female Type of dementia: Alzheimer’s disease Toilet use: Independent Mobility: Independent NPI: Moderately severe Age of the caregiver: 50–59	7	1.197	85.714	4.730
	Gender of the patient: Female Type of dementia: Alzheimer’s disease Feeding: Independent Mobility: Independent NPI: Moderately severe Age of the caregiver: 50–59	7	1.197	85.714	4.730
	Type of dementia: Alzheimer’s disease Toilet use: Independent Bathing: Independent Mobility: Independent Getting lost: Never getting lost NPI: Moderately severe Age of the caregiver: 50–59	7	1.197	85.714	4.730
	Type of dementia: Alzheimer’s disease Feeding: Independent Bathing: Independent Mobility: Independent Getting lost: Never getting lost NPI: Moderately severe Age of the caregiver: 50–59	7	1.197	85.714	4.730
	Gender of the patient: Female Type of dementia: Alzheimer’s disease Toilet use: Independent Mobility: Independent Getting lost: Never getting lost NPI: Moderately severe Age of the caregiver: 50–59	7	1.197	85.714	4.730
	Gender of the patient: Female Type of dementia: Alzheimer’s disease Feeding: Independent Mobility: Independent Getting lost: Never getting lost NPI: Moderately severe Age of the caregiver: 50–59	7	1.197	85.714	4.730
	Gender of the patient: Female Type of dementia: Alzheimer’s disease Feeding: Independent Toilet use: Independent Mobility: Independent NPI: Moderately severe Age of the caregiver: 50–59	7	1.197	85.714	4.730
	Type of dementia: Alzheimer’s disease Feeding: Independent Toilet use: Independent Bathing: Independent Mobility: Independent Getting lost: Never getting lost NPI: Moderately severe Age of the caregiver: 50–59	7	1.197	85.714	4.730
	Gender of the patient: Female Type of dementia: Alzheimer’s disease Feeding: Independent Toilet use: Independent Mobility: Independent Getting lost: Never getting lost NPI: Moderately severe Age of the caregiver: 50–59	7	1.197	85.714	4.730
20	CDR: Moderate dementia Toilet use: Independent Mobility: Independent Getting lost: Never getting lost Employment: Unemployed Type of primary care: Sole caregiver Frequency of care: ≥ 6 days per week	7	1.197	85.714	4.730
	CDR: Moderate dementia Toilet use: Independent Mobility: Independent Getting lost: Never getting lost Caregiver’s marital status: Married Employment: Unemployed Type of primary care: Sole caregiver Frequency of care: ≥ 6 days per week	7	1.197	85.714	4.730
	CDR: Moderate dementia Feeding: Independent Toilet use: Independent Mobility: Independent Getting lost: Never getting lost Employment: Unemployed Type of primary care: Sole caregiver Frequency of care: ≥ 6 days per week	7	1.197	85.714	4.730
	CDR: Moderate dementia Feeding: Independent Toilet use: Independent Mobility: Independent Getting lost: Never getting lost Caregiver’s marital status: Married Employment: Unemployed Type of primary care: Sole caregiver Frequency of care: ≥ 6 days per week	7	1.197	85.714	4.730

*CDR *Clinical dementia rating, *NPI *Neuropsychiatric inventory

The third general rule summarized two rules which showed that the caregiver had a moderate to severe burden when the caregiver shared caregiving with a hired (foreign) caretaker to take care of a female dementia patients with moderately severe NPI. The fourth general rule containing 8 rules summarized that the caregiver had a moderate to severe burden when the caregiver cared for a female patient with moderate dementia along with moderately severe NPI who could mobile independently. The fifth general rule combined 4 rules which reported that the caregiver had a moderate to severe burden when the caregiver was employed to take care of the patient with moderate dementia and moderately severe NPI who could mobile independently.

Two rules were combined to form the sixth general rule. The rule showed that when the caregiver shared caregiving with a hired (foreign) caretaker who cared for the female dementia patient with moderately severe NPI for ≥ 6 days per week, the caregiver felt a moderate to severe burden. A total of 12 rules were summarized into the seventh general rule. The rule reported that the caregiver had a moderate to severe burden when the dementia patient whose age was 75–84 years with moderately severe NPI was cared for by the caregiver whose age was 50–59 years along with the patient was either female or never getting lost. The eighth general rule containing 8 rules reported that when the caregiver was employed with the age of 50–59 years who cared for the dementia patient with moderately severe NPI for ≥ 6 days per week, the caregiver felt a moderate to severe burden. A total of 23 rules were combined to form the ninth general rule. The caregiver had a moderate to severe burden when the caregiver was employed with the age of 50–59 years to care for the dementia patient with moderately severe NPI along with the patient was either female or never getting lost. The tenth general rule summarized 12 rules which showed that the caregiver had a moderate to severe burden when the caregiver was employed and other relative to the patient and shared caregiving with a hired (foreign) caretaker who cared for the patient with AD with the age of ≥ 85 along with the patient either could bath independently or was female.

Four rules were combined to form eleventh general rule. The rule showed that when the married caregiver whose age was 50–59 years cared for the female patient whose age was ≥ 85 years with mild dementia who could mobile independently, the caregiver had a moderate to severe burden. The twelfth general rule containing 4 rules concluded that the caregiver felt a moderate to severe burden when the caregiver was employed and other relative to the patient and shared caregiving with a hired (foreign) caretaker to care for a female patient with ≥ 85 years whose could bath independently. The thirteenth general rule consisting of 12 rules reported that the caregiver had a moderate to severe burden when the caregiver was a child whose age was 50–59 years who cared for the patient whose age was 75–84 years with moderately severe NPI along with the patient was either female or never getting lost. The fourteenth general rule including 5 rules showed that the caregiver felt a moderate to severe burden when the patient whose age was ≥ 85 years had moderately severe NPI who could bath independently and was never getting lost. The fifteen general rule had 4 rules and characterized that when the caregiver was a child who cared for a female patient whose age was 75–84 years with moderately severe NPI who could eat independently, the caregiver had a moderate to severe burden.

The sixteen general rule containing 3 rules showed that when the caregiver cared for the patient whose age was ≥ 85 years with moderately severe NPI for ≥ 6 days per week who could use toilet independently and was never getting lost along with the patient could either bath independently or eat independently, the caregiver had a moderate to severe burden. The seventeenth general rule was based on 11 rules which characterized the caregiver had a moderate to severe burden when the caregiver whose age was 50–59 years cared for the patient with moderately severe NPI and independent mobility along with a combination of the patient with independent bathing and never getting lost, female patient and the patient with never getting lost, female patient and the patient with independent feeding, and the like. The eighteenth general rule had 4 rules which showed that the caregiver had a moderate to severe burden when the caregiver was married with the age of 50–59 years who solely cared for the patient whose age was ≥ 85 and who could use toilet independently. Eleven rules were formed to become the nineteenth general rule. The rule reported that the caregiver felt a moderate to severe burden when the caregiver was aged 50–59 years who cared for the patient with AD who had moderately severe NPI and could mobile independently along with a combination of the patient with independent bath and was never getting lost, the patient was female and never getting lost, and the like. The twentieth general rule consisting of 4 rules showed that the caregiver had a moderate to severe burden when the caregiver was unemployed who solely cared for the patient with moderate dementia for ≥ 6 days per week, and the patient could use toilet independently and mobile independently and was never getting lost.

In Table 3, the caregiving burden was moderate to severe. There was no any rule showing a severe caregiving burden. To further explore the rules containing a severe burden, the minimum confidence was reduced from 85% to 65%. There were sixteen rules found with a severe caregiving burden, which can be further summarized into one general rule as shown in Table 4. When the caregiver was a spouse who solely cared for the male patient with moderate AD, the caregiver felt a severe burden.

**Table 4:** One general rule consisting of sixteen rules for caregivers with a severe burden

Rule no.	Antecedent	No. of Cases	Support (%)	Confidence (%)	Lift
1	Gender of the patient: Male Type of dementia: Alzheimer’s disease CDR: Moderate dementia Relation to the patient: Spouse Type of primary care: Sole caregiver	6	1.026	66.667	21.667
	Gender of the patient: Male Type of dementia: Alzheimer’s disease CDR: Moderate dementia Bathing: Independent Relation to the patient: Spouse Type of primary care: Sole caregiver	6	1.026	66.667	21.667
	Gender of the patient: Male Type of dementia: Alzheimer’s disease CDR: Moderate dementia Relation to the patient: Spouse Type of primary care: Sole caregiver Frequency of care: ≥ 6 days per week	6	1.026	66.667	21.667
	Gender of the patient: Male Type of dementia: Alzheimer’s disease CDR: Moderate dementia Relation to the patient: Spouse Caregiver’s marital status: Married Type of primary care: Sole caregiver	6	1.026	66.667	21.667
	Gender of the patient: Male Type of dementia: Alzheimer’s disease CDR: Moderate dementia Feeding: Independent Relation to the patient: Spouse Type of primary care: Sole caregiver	6	1.026	66.667	21.667
	Gender of the patient: Male Type of dementia: Alzheimer’s disease CDR: Moderate dementia Bathing: Independent Relation to the patient: Spouse Type of primary care: Sole caregiver Frequency of care: ≥ 6 days per week	6	1.026	66.667	21.667
	Gender of the patient: Male Type of dementia: Alzheimer’s disease CDR: Moderate dementia Bathing: Independent Relation to the patient: Spouse Caregiver’s marital status: Married Type of primary care: Sole caregiver	6	1.026	66.667	21.667
	Gender of the patient: Male Type of dementia: Alzheimer’s disease CDR: Moderate dementia Feeding: Independent Bathing: Independent Relation to the patient: Spouse Type of primary care: Sole caregiver	6	1.026	66.667	21.667
	Gender of the patient: Male Type of dementia: Alzheimer’s disease CDR: Moderate dementia Relation to the patient: Spouse Caregiver’s marital status: Married Type of primary care: Sole caregiver Frequency of care: ≥ 6 days per week	6	1.026	66.667	21.667
	Gender of the patient: Male Type of dementia: Alzheimer’s disease CDR: Moderate dementia Feeding: Independent Relation to the patient: Spouse Type of primary care: Sole caregiver Frequency of care: ≥ 6 days per week	6	1.026	66.667	21.667
	Gender of the patient: Male Type of dementia: Alzheimer’s disease CDR: Moderate dementia Feeding: Independent Relation to the patient: Spouse Caregiver’s marital status: Married Type of primary care: Sole caregiver	6	1.026	66.667	21.667
	Gender of the patient: Male Type of dementia: Alzheimer’s disease CDR: Moderate dementia Bathing: Independent Relation to the patient: Spouse Caregiver’s marital status: Married Type of primary care: Sole caregiver Frequency of care: ≥ 6 days per week	6	1.026	66.667	21.667
	Gender of the patient: Male Type of dementia: Alzheimer’s disease CDR: Moderate dementia Feeding: Independent Bathing: Independent Relation to the patient: Spouse Type of primary care: Sole caregiver Frequency of care: ≥ 6 days per week	6	1.026	66.667	21.667
	Gender of the patient: Male Type of dementia: Alzheimer’s disease CDR: Moderate dementia Feeding: Independent Bathing: Independent Relation to the patient: Spouse Caregiver’s marital status: Married Type of primary care: Sole caregiver	6	1.026	66.667	21.667
	Gender of the patient: Male Type of dementia: Alzheimer’s disease CDR: Moderate dementia Feeding: Independent Relation to the patient: Spouse Type of primary care: Sole caregiver Caregiver’s marital status: Married Frequency of care: ≥ 6 days per week	6	1.026	66.667	21.667
	Gender of the patient: Male Type of dementia: Alzheimer’s disease CDR: Moderate dementia Feeding: Independent Bathing: Independent Relation to the patient: Spouse Type of primary care: Sole caregiver Caregiver’s marital status: Married Frequency of care: ≥ 6 days per week	6	1.026	66.667	21.667

## Discussion

The present study delineated the caring scenario that associated with a high caregiving burden in patients with dementia who lived in the community and were relatively well supported. Moderately severe BPSD was the most frequent factor among the caring scenario generated by the Apriori algorithm. Global cognitive function, high care load, and female gender were also associated with a high caregiving burden, while specific ADL domain dysfunction and getting lost were not. When a wife solely took care of her husband with moderate AD who still could eat and bath independently with the frequency of ≥ 6 days per week, she had a severe burden. Previous systematic reviews also found a female spouse with a high care load were risk factors of a high caregiving burden [2, 13, 42]. Our previous studies also revealed that a female spouse caregiver with a high caregiving intensity expressed a moderate or high caregiving burden [6, 16]. Gender distinctions on caregiving burden may have been influenced by the intersection with other socio-demographic factors [42]. Borsje et al. [43] reported that female or spouse caregivers had higher levels of psychological distress compared to male or non-spouse caregivers, therefore, they may express a more severe subjective burden. In addition, nearly 100% of the spouse caregivers were cohabitating with PLWD, which might result in a heavier care load [2]. Several studies have reported that the care load including care duration and intensity (time spending on caregiving per week) were associated with a caregiving burden [2, 13, 44]. The severity of cognitive functions and disease duration were also known as risk factors of a high caregiving burden [5, 44, 45]. The present study highlighted female spouse caregivers without caring helper needed a careful evaluation and an appropriate care plan to relive their care distress or care load. Dementia collaborative care team in our hospital formed care need lists through a face-to-face interview to introduce suitable care resources for patient/caregiver dyads based on their care needs [20]. 

This study found twenty general rules associated with a moderate to severe caregiving burden. Moderately severe BPSD was the most frequent factors among the generated rules (15 out of 20 general rules). In contrast, specific ADL functions, including bathing, toilet use, feeding, and mobility, were usually preserved in the clinical scenarios. Longitudinal studies found that severer BPSD and more rapid functional decline heightened the risk of persisted a high burden level [13, 46, 47]. Few studies analyzed the correlation between behavioral or functional status and caregiving burden and found that BPSD might be more important than functional decline [5, 48]. Machnicki et al. [5] reported that neuropsychiatric symptoms had two times higher strength of association with caregiving burden than function-cognition status. Mohamed et al. [48] concluded that the changes of BPSD severity were correlated with the changes in burden measures, while the changes of ADL functions had limited relationships with the changes in burden measures. Moreover, systematic review articles summarized that BPSD was the most frequent reported and the most predictive factor of caregiving burden and depression regardless of dementia diagnosis [2, 13, 49, 50]. A possible reason to explain that ADL dysfunction was not associated with a high caregiving burden was that only specific ADL dysfunction was analyzed rather than the composite ADL scores or the decline speed. Compared with the NPI total score, using specific ADL domains may weaken the effect of ADL dysfunctions on caregiving burden. Regular evaluations of BPSD and introducing suitable management strategies, such as collaborative care model intervention [51] or the use of encouragement as communication skills [45]are important to alleviate the severity of BPSD and decrease caregiving burden.

Another frequent factor occurring in rules associated with a moderate to severe caregiving burden is caregiver’s employment. Employment and caregiving burden have a reciprocal interaction with each other. Employment is a potential predictor to increase burden when the caregiver takes care of dementia patients [50]and a higher caregiving burden is also associated with a decrease in work productivity [52]. Caregivers who cared for PLWD had higher rates of absenteeism, presenteeism, and overall work impairment when compared to non-caregiving workers [53, 54]. Family care leave, flexible working hours, and transferring suitable caring resources may help employed caregivers to decrease their burden [38]. 

Our study showed that 6.5% and 14% of PLWD reported the history of getting lost or tend to getting lost, respectively. However, all rules generated by the Apriori algorithm did not indicate getting lost was associated with a moderate to severe caregiving burden. Kwok et al. [17] reported about a half of caregivers experienced significant degrees of psychological disturbances after the incident of getting lost. One of possible explanations why the presence of getting lost was not incorporated into rules with a high caregiving burden was a relatively lower percentage of the history of getting lost in our study populations. A previous report in Taiwan found 70% of psychiatric inpatient with AD had the history of getting lost [55]. However, our prevalence rate was compatible with the rate (7.5%) from a community survey in Hong Kong [17]. Another possible explanation was that the dementia patient with the incidence of repeated getting lost was not as high as with the occurrence of BPSD or necessity of functional assistance. The previous report showed that most of the patients (76.8%) experienced only once in the history of getting lost [17]. Getting lost was associated with injury and hospital admission rate [56], appropriate care facilities such as global positioning system (GPS) tracking devices should be introduced to PLWD with the potential of getting lost [57]. 

When considering caring scenarios without moderately severe BPSD (general rules 10, 11, 12, 18, and 20), caregivers taking care of oldest old PLWD (> 85 years old) and having a high care intensity (such as sole or two caregivers taking care more than 6 days per week) were associated with a moderate to severe caregiving burden. Sole caregiver had a higher risk to have a moderate and increasing burden during follow up [47]. In the oldest old population with and without dementia, a widening gap in disability was observed with dementia population tend to experienced functional decline [58]. Increased comorbidities in dementia population were also associated with mobility disability [58]. The care needs in dementia patients with multiple comorbidities maybe different and should comprehensively assessed [22]. Healthcare providers should introduce strategies to maintain functioning, transfer to suitable care resources, and build up caregivers’ caring capacity and educate them how to cope with their own feelings [59]. 

Female patients have been identified among several general rules associated with a moderate to severe caregiving burden. Previous studies showed that BPSD may have sex differences. Meta-analysis found female sex with AD was associated with a higher prevalence and greater severity of several specific neuropsychiatric symptoms, including depression, delusions, and aberrant motor behavior [60]. A cross-sectional study in Taiwan also concluded that delusion and disinhibition were more common in female AD patients [61]. Females with AD also had a faster rate of cognitive decline [62]which was a known risk factor of a high caregiving burden [47]. The association between female gender and BPSD or cognitive decline may partly explain why female patients were occasionally linked into caring scenarios in the present study.

The strength of the present study was to display several caring scenarios indicating a moderate to severe burden in caregivers of PLWD by using a data mining approach. Compared with our previous reports [6, 14, 16], ADL and severity of BPSD were taken into consideration. This study still had some limitations. First, caregivers’ psychiatric symptoms were not analyzed, which were risk factors of a high caregiving burden [2]. Second, the present study used a cross-sectional design which made it impossible to determine causal relationships between correlates and caregivers’ burden or to elucidate the long-term effect of factors on a caregiving burden. Third, because of the heterogeneity between patients’ and caregivers’ factors and the level of caregiving burden, the confidence and support values were set to 85% and 1%, respectively, in order to find association rules. There is no universal approach to set up support and confidence values. In general, a higher confidence value, such as 90% or above, is recommended. However, only eight rules, which were further classified into the first general rule in Table 3, could above 90%, which might weaken the conditional probability of associations in the current study. Fourth, this study analyzed specific ADL dysfunction (bathing, feeding, toilet use, and mobility) but did not use composed ADL scores, which may weaken the effect of ADL dysfunctions on caregiving burden. Fifth, only accompanied caregiver’s burden was evaluated but whether the accompanied caregiver lived with the patient was not known. Thus, only 585 out of 2120 patients had complete data for analysis. In fact, co-living with patients with dementia was also a factor to predict a high caregiving burden [47]. Finally, caregiving is a very complex phenomenon and it is recommended to use different data (such as different hospitals and countries) to find rules emerged from the dataset.

## Conclusions

This study concluded that the severity of BPSD, severity of cognition, patients’ and caregivers’ gender, relation to the care recipient, employment status, and care load were associated with a moderate or severe burden for caregivers who cared for PLWD. The highest caregiving burden was occurred when a female caregiver solely took care of her spouse who had moderate AD. Healthcare providers should provide caregivers’ training about disease knowledge, care strategies toward BPSD and the prevention of cognitive or functional decline, and methods on how to arrange a satisfied leisure and relieve caregivers’ stress. In addition, referring appropriate resources including caregivers’ supportive group or care facilities are necessary especially for the populations with a high risk of a moderate or even severe caregiving burden.

## Supplementary Information

Supplementary Material 1.

## Data Availability

The datasets used and/or analyzed during the current study are available from the corresponding author on reasonable request.

## Abbreviations

AD: Alzheimer’s disease
ADL: Activities of daily living
BPSD: Behavior and psychiatric symptoms of dementia
CCH: Changhua Christian Hospital
CDR: Clinical dementia rating
DLB: Dementia with Lewy bodies
NIA-AA: The National Institute on Aging-Alzheimer’s Association
NPI: Neuropsychiatric Inventory
PLWD: People living with dementia
VASCOG: Vascular Behavioral and Cognitive Disorders
ZBI: the Zarit burden interview
