# Supplementary material for: Caregivers’ burden analytics: combining variables from patients with dementia and their caregivers
Source: BMC Geriatr. 2025 Aug 14;25:620. doi: 10.1186/s12877-025-06284-y (PMC12351875; doi:10.1186/s12877-025-06284-y)
Supplement: Supplementary file 1 — Supplementary Material 1. [file 12877_2025_6284_MOESM1_ESM.doc]

STROBE Statement—Checklist of items that should be included in reports of ***cross-sectional studies***

|  | Item No | Recommendation |
| --- | --- | --- |
| **Title and abstract** | 1 | **Caregivers’ Burden Analytics: Combining Variables from Patients with Dementia and their Caregivers** |
|  |  | No similar studies were found to combine variables from patients and their caregivers. |
| Introduction | | |
| Background/rationale | 2 | Factors associated with a caregiving burden are complex and vary with time since burden is a subjective measure influenced by physical, economic, psychosocial strain and has an interaction among caregiver resources, vulnerabilities, and care demands.^12^ Previous systematic reviews reported that no uniform model for predicting the trajectory of caregiving burden^13^, but severer BPSD and functional decline were factors reported frequently and should be considered as important factors to be associated with a high caregiving burden.^2^ Although fewer reports support, types of dementia, caregiver’s socio-demographical and psychological factors, cohabitation, and kinship within the dyads were also possible important factors.^2,13^ A previous cohort study followed 18 months found that patients with Lewy body disease, the severer dementia stage or BPSD, and caregiver depression were associated with a high caregiving burden.^4^  To delineated caring scenario associated with a high burden for caregivers who care for PLWD, we used the Apriori algorithm, a data miming method, from a multidimensional viewpoint. Our previous study which included patients’ and caregivers’ basic characteristics and care load concluded that when a wife solely took care of her husband living with dementia, who was 75-79 years old and still could walk independently, she would experience a high caregiving burden.^6^ Another study further included caregivers’ psychiatric conditions and found the following scenarios were associated with a moderate to severe burden: caregivers expressed any one of the mood (emotional liability, depressive, or anxious) and patients still could walk independently.^14^ The presence of specific neuropsychiatric symptoms were associated with varying degrees of caregiving burden in female patients with Alzheimer’s disease (AD), for example, crying spells and aggression led to a moderate to severe burden.^15^ In patients with vascular cognitive impairment, an employed female caregiver who took care of her spouse for ≥6 days per week and helped with all key activities was likely to experience a moderate to severe burden.^16^  Severer BPSD and worsen activities of daily living (ADL) function were important factors associated with a high caregiving burden.^2^ Their significance may also be different. Machnicki et al.^5^ reported that behavioral symptoms had more than two times higher strength of an association than functional decline on caregiving burden. Studies discussing spatial disorientation and caregiving burden were relatively limited. Previous report showed that caregivers experienced significant degrees of psychological disturbances after the incident of getting lost.^17^ Our previous reports did not include the severity of BPSD, spatial disorientation, and items of ADL to form caring scenarios. |
| Objectives | 3 | Therefore, the aim of the present study was to elucidate the combination of patients’ and caregivers’ factors, especially BPSD severity and specific ADL items impairment, with a moderate or high caregiving burden by using the Apriori algorithm. |
| Methods | | |
| Study design | 4 | Patients who were diagnosed with dementia at the memory clinic of Changhua Christian Hospital (CCH) from January 2015 to July 2021 were enrolled in this study.  Initially, there were 2120 patients but only 585 patients had complete data for analysis. To better reflect the conditions for patients with dementia and their caregivers in a timely basis, the most recent diagnosis and interview data were used.  In this study, the variables from patients with dementia such as age, gender, type of dementia, CDR, feeding, toilet use, bathing, mobility, getting lost, and neuropsychiatric inventory (NPI) were provided in Table 1.  The variables from caregivers were composed of age, relation to the patient, marital status, employment, type of primary care, frequency of care, and caregiving burden as shown in Table 2. |
| Setting | 5 | Please refer to Study design |
| Participants | 6 | Patients who were diagnosed with dementia at the memory clinic of Changhua Christian Hospital (CCH) from January 2015 to July 2021 were enrolled in this study. For each patient, the diagnosis of dementia was evaluated by a clinical psychologist based on the clinical dementia rating (CDR) scale.^18^  Initially, there were 2120 patients but only 585 patients had complete data for analysis. |
| Variables | 7 | In this study, the variables from patients with dementia such as age, gender, type of dementia, CDR, feeding, toilet use, bathing, mobility, getting lost, and neuropsychiatric inventory (NPI) were provided in Table 1.  The variables from caregivers were composed of age, relation to the patient, marital status, employment, type of primary care, frequency of care, and caregiving burden as shown in Table 2. |
| Data sources/ measurement | 8* | The attending physicians at CCH memory clinic invited the dementia patients who still lived in the community and their caregivers to join the case manager-centered dementia collaborative care model.^19^ More than 90% of patients diagnosed with dementia were enrolled in the model. The care team consisted of various professionals, including physicians, psychologists, social workers, dieticians, occupational therapists, pharmacists, and nursing case managers. After joining the care model, face-to-face evaluations regarding to ADL function, BPSD, living status and care modes, caring problems, and caregiving burden were conducted every 6 months to identify unmet care needs and provide relevant interventions.^20-22^ For caregiving burden, only the family caregiver who accompanied the patient to the clinic was evaluated. If more than one family caregivers visited, the primary caregiver’s burden was assessed. All data were recorded in the electronic charts.  Dementia severity were categorized as very mild (CDR = 0.5), mild (CDR = 1), moderate (CDR = 2), and severe (CDR = 3) stages. Dementia subtypes were diagnosed and classified in accordance with different guidelines. AD was corresponded to the National Institute on Aging-Alzheimer’s Association (NIA-AA)^23,24^ whereas the diagnostic criteria for vascular cognitive impairment were from the International Society for Vascular Behavioral and Cognitive Disorders (VASCOG).^25^ The diagnosis of Parkinson’s disease dementia or dementia with Lewy bodies (DLB) followed the Movement Disorder Society-Task force criteria and the fourth consensus report of the DLB Consortium.^26,27^ The functional assessment including bathing, feeding, toilet use, and mobility were created by the CCH dementia center. Some patients with dementia developed troublesome ADL behaviors such as defecate indiscriminately, need remind, or even pressurized to perform bathing. Case managers performed the ADL evaluation and provided relevant care plans during the assessment.  The caregiving burden was evaluated by the Zarit burden interview with four categories, i.e., little or no burden (0-20 points), mild to moderate burden (21-40 points), moderate to severe burden (41-60 points), and severe burden (61-88 points).^29^  For caregiving burden, Only the family caregiver who accompanied the patient to the clinic was evaluated. If more than one family caregivers who took care of the patient, the primary caregiver’s burden was assessed. When a caregiving burden falls in a particular category, a value of 1 is assigned, and a value of zero is given to the other three categories. |
| Bias | 9 | Not applicable. |
| Study size | 10 | Not applicable. |
| Quantitative variables | 11 | Use the original scale to perform major analysis. |
| Statistical methods | 12 | The Apriori algorithm was employed. |
|  |  | Not applicable. |
|  |  | Remove the missing data entirely. |
|  |  | Not applicable. |
|  |  | Not Applicable. |
| Results | | |
| Participants | 13* | Not applicable. |
|  |  | Not applicable. |
|  |  | Not applicable. |
| Descriptive data | 14* | In this study, the variables from patients with dementia such as age, gender, type of dementia, CDR, feeding, toilet use, bathing, mobility, getting lost, and neuropsychiatric inventory (NPI) were provided in Table 1.  The variables from caregivers were composed of age, relation to the patient, marital status, employment, type of primary care, frequency of care, and caregiving burden as shown in Table 2. |
|  |  | Not applicable. |
| Outcome data | 15* | Report numbers of outcome events or summary measures |
| Main results | 16 | Not applicable. |
|  |  | Not applicable. |
|  |  | Not applicable. |
| Other analyses | 17 | Not applicable. |
| Discussion | | |
| Key results | 18 | The present study delineated the clinical conditions that associated with a high caregiving burden in patients with dementia who lived in the community and were relatively well supported. Global cognitive function, high care load, female gender as well as moderately severe BPSD were associated with a high caregiving burden than specific ADL domain dysfunction. |
| Limitations | 19 | This study still had some limitations. First, caregivers’ psychiatric symptoms were not analyzed, which were risk factors of a high caregiving burden.^2^ Second, the present study used a cross-sectional design which made it impossible to determine causal relationships between correlates and caregivers’ burden or to elucidate the long-term effect of factors on a caregiving burden. Third, because of the heterogeneity between patients’ and caregivers’ factors and the level of caregiving burden, the confidence and support values were set to 85% and 1%, respectively, in order to find association rules. There is no universal approach to set up support and confidence values. In general, a higher confidence value, such as 90% or above, is recommended. However, only eight rules, which were further classified into the first general rule in Table 3, could above 90%, which might weaken the conditional probability of associations in the current study. Fourth, this study analyzed specific ADL dysfunction (bathing, feeding, toilet use, and mobility) but did not use composed ADL scores, which may weaken the effect of ADL dysfunctions on caregiving burden. Fifth, only accompanied caregiver’s burden was evaluated but whether the accompanied caregiver lived with the patient was not known. In fact, co-living with patients with dementia was also a factor to predict a high caregiving burden.^43^ Finally, caregiving is a very complex phenomenon and it is recommended to use different data (such as different hospitals and countries) to find rules emerged from the dataset. |
| Interpretation | 20 | Not applicable. |
| Generalisability | 21 | Not applicable. |
| Other information | | |
| Funding | 22 | Not applicable. |

*Give information separately for exposed and unexposed groups.

**Note:** An Explanation and Elaboration article discusses each checklist item and gives methodological background and published examples of transparent reporting. The STROBE checklist is best used in conjunction with this article (freely available on the Web sites of PLoS Medicine at http://www.plosmedicine.org/, Annals of Internal Medicine at http://www.annals.org/, and Epidemiology at http://www.epidem.com/). Information on the STROBE Initiative is available at www.strobe-statement.org.
